# Superior Recognition Performance for Happy Masked and Unmasked Faces in Both Younger and Older Adults

**DOI:** 10.3389/fpsyg.2012.00520

**Published:** 2012-11-30

**Authors:** Joakim Svärd, Stefan Wiens, Håkan Fischer

**Affiliations:** ^1^Aging Research Center, Karolinska InstituteStockholm, Sweden; ^2^Department of Psychology, Stockholm UniversityStockholm, Sweden

**Keywords:** emotion, faces, aging, masking, happy, fearful, positivity bias, neuroticism

## Abstract

In the aging literature it has been shown that even though emotion recognition performance decreases with age, the decrease is less for happiness than other facial expressions. Studies in younger adults have also revealed that happy faces are more strongly attended to and better recognized than other emotional facial expressions. Thus, there might be a more age independent happy face advantage in facial expression recognition. By using a backward masking paradigm and varying stimulus onset asynchronies (17–267 ms) the temporal development of a happy face advantage, on a continuum from low to high levels of visibility, was examined in younger and older adults. Results showed that across age groups, recognition performance for happy faces was better than for neutral and fearful faces at durations longer than 50 ms. Importantly, the results showed a happy face advantage already during early processing of emotional faces in both younger and older adults. This advantage is discussed in terms of processing of salient perceptual features and elaborative processing of the happy face. We also investigate the combined effect of age and neuroticism on emotional face processing. The rationale was previous findings of age-related differences in physiological arousal to emotional pictures and a relation between arousal and neuroticism. Across all durations, there was an interaction between age and neuroticism, showing that being high in neuroticism might be disadvantageous for younger, but not older adults’ emotion recognition performance during arousal enhancing tasks. These results indicate that there is a relation between aging, neuroticism, and performance, potentially related to physiological arousal.

## Introduction

In the aging and emotion literature it is often assumed that, although recognition performance for facial expressions seems to decrease with age, the absolute performance level for happy faces is preserved (see Isaacowitz et al., 2007, p. 148 for a comprehensible table). However, results from a recent meta-analysis of aging and emotion recognition (Ruffman et al., 2008) showed a decrease in performance with age also in recognition of happy faces. Even though older adults performed worse than younger adults in recognition of all facial expressions except for faces displaying disgust, the age-related differences in performance were smaller for happy (and surprised) faces than for other facial expressions. Thus, recognition performance for happy faces seems to be relatively preserved in older adults, which is in line with the notion of a shift of focus toward positive, and away from negative information with advancing age (e.g., Mather and Carstensen, 2005).

On the other hand, a growing literature based on younger adults shows an advantage for happy faces compared to other facial expressions both in recognition and attention. For example, Calvo and Lundqvist (2008) found that during a free-viewing condition, accuracy rates were higher and reaction times were shorter for happy faces compared to all other emotional expressions. When faces were presented for shorter durations (25–500 ms), recognition of happy faces reached a ceiling at the 50 ms duration. No other expressions reached the ceiling even at the 500 ms duration. Further, visual search studies with younger adults showed that, when real faces were used as targets, happy faces were detected faster and with higher accuracy than other emotional faces (Juth et al., 2005; Calvo and Nummenma, 2008; Calvo and Marrero, 2009). The fact that this happy face advantage is evident even outside of overt visual attention and for inverted faces has led to the conclusion that it is driven by processing of salient facial features (Calvo et al., 2010). Thus, rather than positive preferences in older adults (e.g., Mather and Carstensen, 2005), the relative preserved recognition performance for happy faces might be explained by a more general advantage for happy faces that is independent of age (e.g., Calvo et al., 2010).

Although the happy face advantage has been investigated in several spatial attention studies (e.g., Juth et al., 2005; Calvo and Marrero, 2009), the temporal aspect of the happy face advantage is relatively unexplored. Calvo and Lundqvist (2008) found that in overt recognition, younger adults reached the ceiling in performance already when faces were displayed for 50 ms. However, the accuracy was higher for happy faces than all other facial expressions in the shortest presentation time condition (25 ms). Thus, the onset of the happy face advantage in recognition performance remains unclear. By using a backward masking paradigm with stimulus onset asynchronies (SOAs) starting at 17 ms, we investigated when the happy face advantage occurred during visual processing. This technique allows parametrical modulation of the visibility that, in turn, allows investigation of recognition performance as a continuum from low to high levels of visibility. In addition to making it possible to study the temporal aspect of the happy face advantage, the present masked recognition task also differs from other recognition tasks, in which participants are asked to label the facial expressions (McDowell et al., 1994; Phillips et al., 2002; Calder et al., 2003). In such recognition tasks, participants typically have several negative but only one positive expression to choose from. The present masked recognition task used a forced choice task, where participants could choose between fearful, happy, or neutral expressions. Such distinct facial expressions in terms of emotional valence minimize the risks of confusing expressions. Thus, our masked recognition task gives a valid measure of processing more clear-cut positive and negative emotional information in contrast to non-forced recognition tasks where participants have several negative expressions to choose from which might lead to confusion, especially among older adults (Ruffman et al., 2008).

In addition, and with a quite different research question, we investigated the effect of neuroticism on processing of briefly presented emotional stimuli. The rationale for this was the assumption of an inverted U-shaped relationship between arousal and performance (Yerkes and Dodson, 1908; Humphreys and Revelle, 1984). In series of visual attention tasks, Szymura and Wodniecka (2003) found that for less demanding conditions, with relatively long stimuli presentations (850 ms), neurotics, and controls (younger adults) performed equally well. However, in more difficult conditions when the presentation time was shorter, neurotics performed worse than controls. These results may indicate that neurotic individuals’ arousal levels became too high for optimal performance in the more difficult condition, leading to impaired performance. Given results from previous research showing decreased physiological arousal in older adults compared to younger adults when exposed to emotional pictorial stimuli (Gavazzeni et al., 2008) and emotion-eliciting film clips (Tsai et al., 2000), it was of interest to investigate whether the high baseline arousal associated with neuroticism (Eysenck and Eysenck, 1985) would have an age-related reverse influence on emotion recognition performance in the present arousal enhancing backward masking task. Based on Szymura and Wodniecka’s (2003) findings, we expected a decrease in performance of high neurotic younger adults in the mask recognition task, but not in the unmasked recognition task. For older adults, we expected the reverse. An amplified arousal level in high neurotic older adults should be beneficial for their performance. That is, because older adults on average have a lower arousal level during processing of emotional visual information (Tsai et al., 2000; Gavazzeni et al., 2008), high neurotic older adults are unlikely to surpass an optimal arousal level in the current study task. Instead, their arousal levels were expected to approach optimal arousal levels.

To summarize, we used a backward masking paradigm with several SOAs in order to investigate the temporal aspect of the happy face advantage in younger and older adults. This approach allowed an investigation of recognition performance as a continuum from low to high levels of visibility. Lastly, we investigated whether neuroticism and aging has a combined effect on emotion recognition performance during an arousal enhancing condition.

## Materials and Methods

### Participants

The sample consisted of 20 older (10 women and 10 men) and 19 younger participants (10 women and 9 men). Mean age (SD) was 25.5 years (2.6) for younger women, 27.3 years (2.6) for younger men, 72.4 years (2.3) for older women, and 75.0 years (2.6) for older men. An ANOVA with age (years) as dependent variable and age group and sex as independent variables revealed main effects for age groups, *F*(1, 35) = 3446. 59, *p* < 0.001, and sex, *F*(1, 35) = 7.58, *p *= 0.009 (men older than women), but no interaction between age group and sex, *F*(1, 35) < 1, *p *> 0.05. Independent samples *t*-tests showed that there were no differences between younger and older adults in any of the demographic variables (*t*s < 1.7, *p*s > 0.05): years of education (*M*_young_ = 14.4, SD_young_ = 1.9; *M*_old_ = 14.15, SD_old_ = 1.8); Mini mental state exam – MMSE (*M*_young_ = 29.1, SD_young_ = 0.8; *M*_old_ = 28.7, SD_old_ = 1.1; Folstein et al., 1975); Neuroticism (*M*_young_ = 83.8, SD_young_ = 26.3; *M*_old_ = 70.0, SD_old_ = 23.7; Costa and McCrae, 1992); and Vocabulary (*M*_young_ = 24.1, SD_young_ = 3.1; *M*_old_ = 25.1, SD_old_ = 2.7; Dureman, 1960). All subjects had responded to ads regarding participation in psychological studies at the Aging Research Center at Karolinska Institute and were contacted via phone. They were asked if they would like to participate in a study in which pictures of faces would be shown at different durations, and their task was to judge the facial expressions. The study was approved by the local ethics committee. Participation was based on written informed consent and participants received a lottery ticket worth 6 Euro.

For the neuroticism analysis, participants were divided into low and high neurotics using a median split approach. Thus, the analyses contained 39 subjects divided into four groups; younger high neurotics (*n* = 10; *M*_Nscore_ = 103.8, SD_Nscore_ = 20.03), younger low neurotics (*n *= 9; *M*_Nscore_ = 61.56, SD_Nscore_ = 7.32), older high neurotics *(n *= 9; *M*_Nscore_ = 88.89, SD_Nscore_ = 20.06), and older low neurotics *(n *= 11; *M*_Nscore_ = 54.55, SD_Nscore_ = 12.87). Compared to a norm group consisted of 363 men and 269 women (Costa and McCrae, 1985), across sex, the participants did not differ in neuroticism scores, *t*(38) < 1, *p* > 0.05 (*M*_norm_ = 78.35; *M*_current study_ = 76.72).

### Materials

Thirty gray scale pictures of faces depicted 10 individuals, each displaying fearful, neutral, and happy facial expressions (Ekman and Friesen, 1976). Half of the identities were female (labeled by Ekman and Friesen as C, MF, NR, PF, and SW), and half were male (EM, GS, JJ, PE, and WF). Thirty masking pictures were generated by scrambling the 30 original pictures. Each original picture (200 × 310 pixels) was divided into blocks of 5 × 5 pixels (62 rows by 40 columns), and the 2480 blocks were shuffled so that their location was random. The experiment was run on a desktop PC with a standard 17-inch cathode ray tube (CRT) monitor. Screen resolution was 800 × 600 pixels, refresh rate was 60 Hz and the experiment was programmed in Presentation 9.3 (Neurobehavioral Systems, www.neurobs.com). Participants viewed pictures at a distance of about 1 m and the visual angle was 7.4° × 9.4°.

### Procedure

The experiment consisted of two tasks; the masked recognition task was preceded by an unmasked recognition and an intensity rating task. The unmasked recognition task enabled comparisons of overt facial expression recognition performance between participants of the present study and participants in previous studies. In the rating task, all faces were shown until participants were done rating intensity and facial expression labeling. In the masked recognition task, the same faces were shown briefly and masked by scrambled versions of the pictures. Piloting showed that older participants tended to be unfamiliar with computers. To avoid distraction from the tasks, none of the participants were required to enter their answers themselves on a keyboard. Instead, participants received screen prompts to respond verbally and their answers were collected by the experimenter (Joakim Svärd).

#### Unmasked recognition and intensity rating task

Participants were informed that they would be shown pictures of faces for unlimited durations, and their task was to rate intensity and to label the emotional facial expression. Participants rated intensity of expressions on a nine-point scale (1 = very low, 9 = very high). Because some pilot participants tended to confuse intensity of expression with accuracy of emotional expression (e.g., a neutral face that was clearly neutral was rated as nine), participants in the present experiment completed a practice task. They were shown line drawings of neutral and emotional faces with different degrees of emotional expression and practiced to judge intensity rather than accuracy of the expression. In the actual task, participants were shown all 30 pictures in random order. While each picture was presented on the screen, participants rated intensity of expression. Next, they labeled the expression by choosing among seven response alternatives (happy, surprised, angry, sad, neutral, disgusted, and fearful) presented on the screen. In order to be able to make comparisons with other overt facial expression recognition studies, we used the seven response alternative approach.

To analyze the recognition data, indexes of signal detection theory (MacMillan and Creelman, 1991) were used to separate discrimination ability from response biases, that is, participants’ tendency to favor one response alternative over the others. For example, if response biases are not taken into account (as for percent correct), participants favoring happy obtain an inflated percent correct for happy faces. To separate discrimination ability (*d*′) from response biases (*C*), signal detection indexes (Snodgrass and Corwin, 1988; MacMillan and Creelman, 1990) were calculated for each participant and target emotion (fearful, happy, neutral) based on *hit rates* (i.e., percent of trials in which a target expression was labeled correctly as a target, e.g., happy face labeled as happy) and *false alarm rates* (i.e., percent of trials in which a non-target expression was labeled incorrectly as a target, e.g., fearful and neutral face labeled as happy). Because signal detection indexes cannot be computed with hit and false alarm rates that are 0 or 1, recommendations were followed (Snodgrass and Corwin, 1988) when calculating each rate; half a trial was added as a response (numerator) and one trial was added to the total number of possible responses (denominator).

For C, positive values reflect conservative responses biases (i.e., the target expression is favored less), negative values reflect liberal biases (i.e., the target expression is favored more), and zero reflects no bias. That is, *C* = 0 is the point at which it is as probable to classify a target as a non-target (*miss*) as it is to classify a non-target as a target (false alarm).

#### Masked recognition task

**Figure 1:**
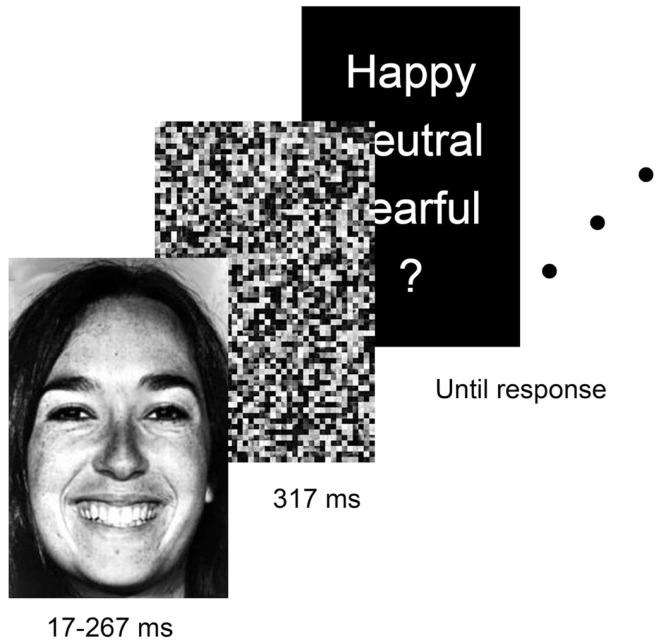
**Example of the stimulus presentation sequence in the masked recognition task**. Target pictures were shown for durations of 17, 33, 50, 67, 83, 117, and 267 ms, followed by a scrambled picture (mask) for 317 ms.

Participants were informed that they would be shown faces displaying fearful, neutral, and happy expressions at various durations. Due to the risk of floor effects that would have followed a seven response choice as used in the unmasked recognition task, we instead used a three response choice, in line with the majority of previous backward masking studies of facial expression recognition (e.g., Duan et al., 2010; Bornemann et al., 2012). On each trial, a picture would be shown followed immediately by another (scrambled) picture (Figure 1). Participants were informed that a face would always be shown, and that they were to decide if the expression was happy, neutral, or fearful. Importantly, to study response biases, participants were not informed that all emotions were equally likely across trials. However, they were informed that picture duration might be sufficiently brief to appear to them as if no face was presented. If so, they were instructed to guess the emotional expression. Target pictures were shown for durations of about 17, 33, 50, 67, 83, 117, and 267 ms, corresponding to 1, 2, 3, 4, 5, 7, and 16 refresh cycles on a 60 Hz monitor, respectively. That is, on CRT monitors, picture duration is a function of number of refresh cycles (Bridgeman, 1998). Because the software synchronized picture display with individual refresh cycles (Wiens et al., 2004), at a refresh rate of 60 Hz, each refresh cycle lasted about 17 ms (1000 ms/60 Hz = 16.7 ms). After each target, a masking picture was shown for 317 ms (19 refresh cycles). The 30 masking pictures were used once on 30 consecutive trials in random order. In total, the task consisted of 210 trials (7 durations × 3 expressions × 10 individual faces). To avoid fatigue, participants were allowed a 5-min break after completing half of the trials.

As for the unmasked recognition task, signal detection analyses (MacMillan and Creelman, 1991) were performed to separate discrimination ability (*d*′) from response bias (*C*). Based on hit and false alarm rates, these indexes were calculated for each participant for each target emotion (fearful, neutral, and happy) and the seven picture durations.

## Results

Although the goal was to study effects of age, the sample size was sufficient to analyze also effects of sex. The reason for including sex as a factor was findings that showed that women seems to be better than men in facial expression recognition during low intensity condition (Hoffman et al., 2010). However, because effects of sex are not the main focus in the present study, results for sex are mentioned but not discussed further.

In all analyses involving repeated measures, regular degrees of freedom are reported together with observed significance levels after Greenhouse–Geisser correction. Results were considered significant at *p* < 0.05.

### Unmasked recognition and intensity rating task

#### Unmasked recognition

When participants labeled the emotional expression of the target faces, they could choose among happy, surprised, angry, sad, neutral, disgusted, or fearful expressions.

**Figure 2:**
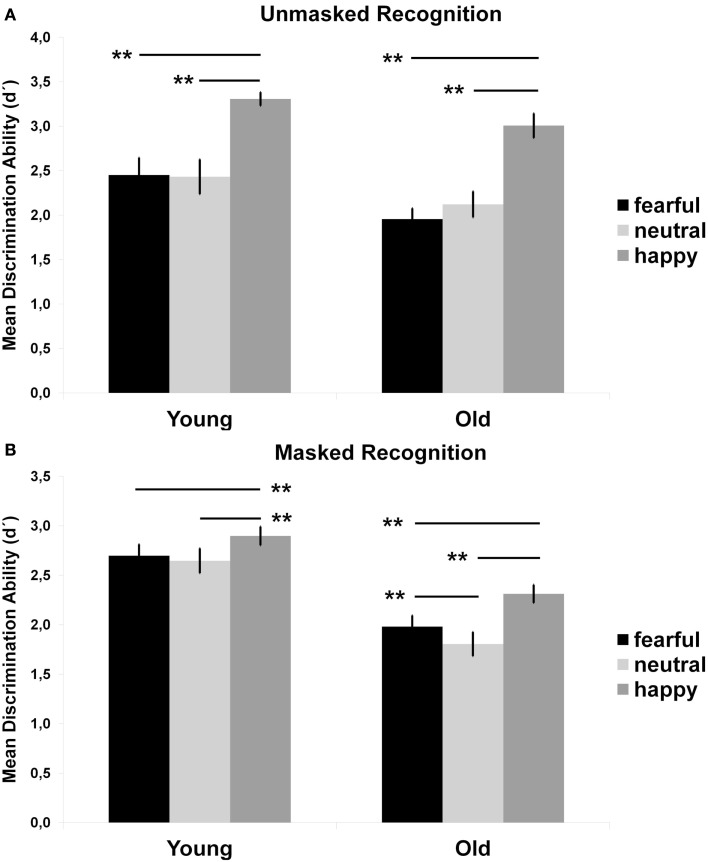
**Mean (SE) recognition performance (*d*′) across durations in the unmasked recognition task (A) and masked recognition task (B), separately for expression and age**. *Note*. Results of *t*-tests for age differences are denoted with asterisks. **p < 0.01, *p < 0.05.

An ANOVA of *d*′ with age, sex, and neuroticism as between-subjects variables and emotion as a within-subjects variable revealed a significant main effect for age, *F*(1, 31) = 8.73, *p* = 0.006. As shown in Figure 2A, younger adults performed better than older adults across emotions. There was also a main effect for emotion, *F*(2, 62) = 46.84, *p* < 0.001. Across age, performance was significantly better for happy faces than for neutral, *F*(1, 31) = 90.12, *p* < 0.001, and fearful faces, *F*(1, 31) = 78.15, *p* < 0.001, and that the latter two did not differ from each other, *F*(1, 31) < 1, *p *> 0.05. For completeness, *t*-tests were computed and showed that younger adults performed better than older adults on fearful faces, *t*(37) = 2.15, *p* = 0.039, but not on happy, *t*(37) = 1.9, *p* = 0.068, or neutral faces, *t*(37) = 1.27, *p* = 0.213 (Table 1). The main effect of sex, *F*(1, 31) = 5.56, *p* = 0.007, showed that women performed better than men (*M*_women_ = 2.74, SE_women _= 0.06; *M*_men_ = 2.29, SE_men_ = 0.06). However, these main effects were qualified by an interaction between age, sex, neuroticism, and emotion, *F*(2, 62) = 3.94, *p* = 0.029. Two follow-up ANOVAs, computed for men and women separately, revealed an emotion by neuroticism by age interaction for men, *F*(2, 30) = 6.56, *p* = 0.004, but not for women, *F*(2, 30) < 1, *p* > 0.05, parsimoniously described as that older low neurotic men performed better than older high neurotic men for neutral faces.

**Table 1:** **Mean (and SDs) intensity ratings and mean *d*′ (and SDs) in the unmasked recognition**.

Task	Younger	Older	*p*
**INTENSITY RATING**			
Fearful	6.74 (1.3)	7.52 (0.9)	**0.04**
Neutral	2.69 (1.8)	4.38 (2.2)	**0.013**
Happy	6.77 (1.2)	6.98 (1.2)	0.581
**UNMASKED RECOGNITION**			
Fearful	2.45 (0.9)	1.96 (0.5)	**0.039**
Neutral	2.43 (0.9)	2.12 (0.7)	0.213
Happy	3.31 (0.3)	3.01 (0.6)	0.068

*Bold p-values indicate significance level of <0.05*.

#### Intensity ratings

Participants rated each of the 10 individuals with fearful, neutral, and happy expressions in intensity of expression on a 9-point scale. Because analyses of participants’ ability to label expressions correctly suggested age and sex differences (below), analyses of intensity ratings are reported only for faces that were labeled correctly. Thus, this analysis does not confound intensity ratings with participants’ ability to label expressions correctly. However, results were comparable when all trials were included.

An ANOVA of intensity ratings with age, sex, and neuroticism as between-subjects variables and facial emotion (fear, happy, neutral) as a within-subjects variable showed significant main effects for emotion, *F*(2, 62) = 117.74, *p* < 0.001, and for age, F(1, 31) = 6.07, *p* = 0.02. However, these main effects were qualified by an interaction between age and facial emotion, *F*(2, 62) = 5.23, *p* = 0.016. Results from follow-up *t*-tests are presented in Table 1.

### Masked recognition task

**Figure 3:**
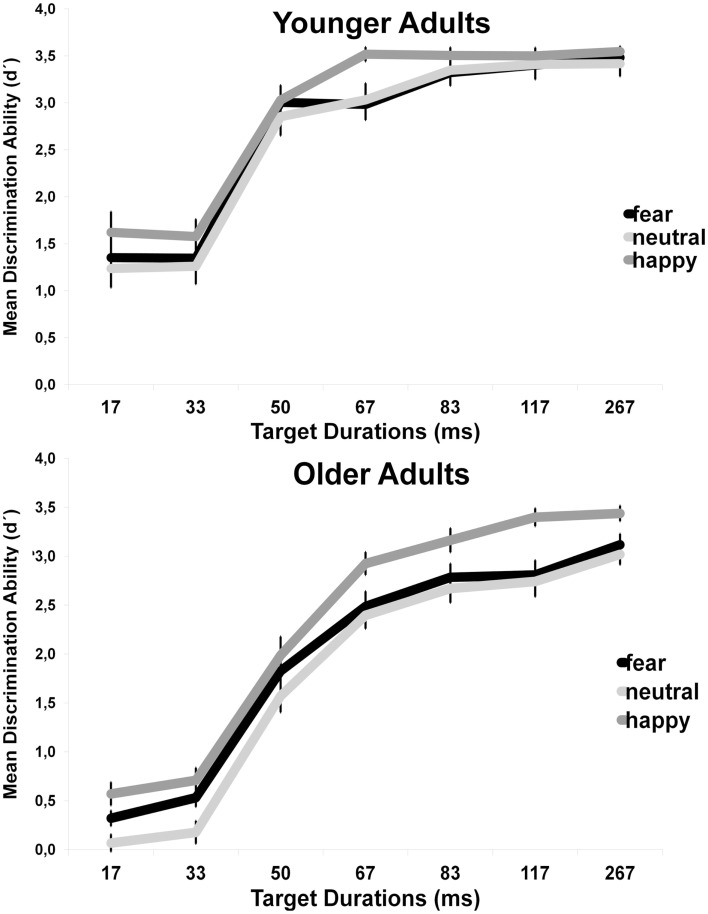
**Mean (SE) recognition performance (*d*′) in the masked recognition task depending on picture duration, facial emotion and age**.

**Figure 4:**
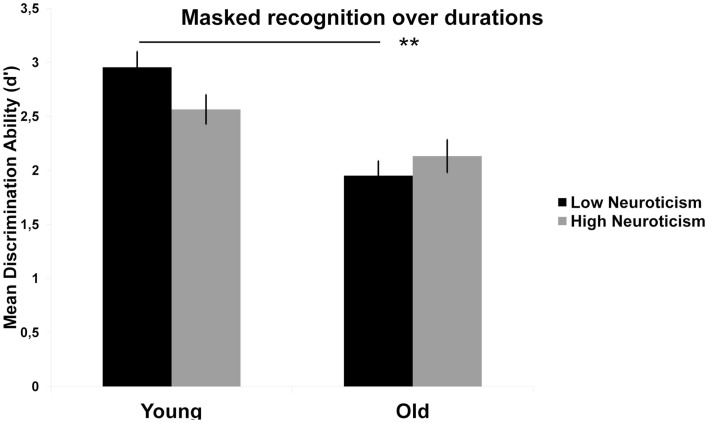
**Mean (SE) recognition performance (*d*′) in the masked recognition task (across emotions and durations) for age and neuroticism**. *Note*. Results of *t*-tests are denoted with asterisks. **p < 0.01, *p < 0.05.

Due to various research questions, the masked recognition performance was computed twice. In the main age and emotion analysis, the aim was to investigate the development of facial expression recognition performance along durations ranging from 17 up to 267 ms. Rather than investigate performance at different durations, the aim in the additional neuroticism analysis was to investigate performance in relation to the different nature of the tasks (i.e., high arousal generating task (masked recognition) versus low arousal generating tasks (unmasked recognition). Because even the longest durations in the current study were beyond the exposure previously reported as aggravating for high neurotic younger adults (Szymura and Wodniecka, 2003) and performance at single durations is not considered important for the neurotic analysis, an additional univariate ANOVA across durations were computed for effects of neuroticism in the masked recognition task. In addition, because we expect arousal rather than emotional valence *per se* to moderate performance, the ANOVA was collapsed across emotions. Thus, for the masked recognition task two separated analyses were computed; age, sex and duration (Figure 3) followed by age, sex and neuroticism across all durations (Figure 4).

#### Recognition performance: age and duration

An ANOVA of *d*′ with age and sex as between-subjects variables and picture duration and emotion as within-subjects variables revealed a significant main effect for age, *F*(1, 35) = 22.11, *p* < 0.001. As shown in Figure 2B, younger adults performed better than older adults across all durations (*M*_young_ = 2.75, SE_young_ = 1.1; *M*_old_ = 2.03, SE_old_ = 1.1). There was also a main effect of emotion, *F*(2, 70) = 57.08, *p* < 0.001. Contrast tests across age showed that performance was significantly better for happy faces (*M*_happy_ = 2.61, SE_happy _= 0.07) than for neutral (*M*_neutral_ = 2.23, SE_neutral _= 0.09), *F*(1, 35) = 90.83, *p* < 0.001, and fearful faces (*M*_fearful_ = 2.34, *SE*_fearful _= 0.08), *F*(1, 35) > 41.88, *p* < 0.001. However, in contrast to results for the unmasked recognition task, performance was better for fearful than neutral faces, *F*(1, 35) = 18.07, *p* < 0.001. Further, these main effects was qualified by a interaction between emotion and age, *F*(2, 70) = 6.19, *p* = 0.006. Contrast tests in two follow-up ANOVAs, separated by age, showed that performance were better for fearful (*M*_oldfear _= 1.98, SE_oldfear_ = 0.11) than for neutral faces (*M*_oldneu _= 1.81, SE_oldfear_ = 0.12) for older, *F*(1, 17) = 24.45, *p* < 0.001, but not younger adults, *F*(1, 17) = 1.62, *p *< 0.05. In both age groups, performance was better for happy faces compared to fearful and neutral faces (*ps* < 0.001). Lastly, there was a main effect of duration, *F*(6, 210) = 341.24, *p *< 0.001, which was qualified by a interaction between duration and age, *F*(6, 210) = 8.7, *p *< 0.001, parsimoniously described as follow: with longer durations, the age-related differences across emotions decreased.

In order to investigate recognition performance for all facial expression, for all durations, both within and between age groups planned paired samples *t*-tests and independent samples *t*-test were conducted (see Table 2).

**Table 2:** **Mean *d*′ (and SDs) for masked recognition performance between and within age groups**.

Duration (ms)	Fearful			Neutral			Happy		
	Y	O	*p*	Y	O	*p*	Y	O	*p*
17	1.35 (0.9)	0.32 (0.4)	**<0.001**	1.24 (0.9)	0.07 (0.4)	**<0.001**	1.62 (1)	0.57 (0.5)	**<0.001**
33	1.35 (0.7)	0.53 (0.4)	**<0.001**	1.26 (0.8)	0.18 (0.5)	**<0.001**	1.58 (0.8)	0.71 (0.6)	**<0.001**
50	3 (0.7)	1.83 (0.8)	**<0.011**	2.85 (0.9)	1.57 (0.8)	**<0.001**	3.03 (0.7)	1.99 (0.9)	**<0.001**
67	3 (0.7)	2.5 (0.7)	**0.036**	3.03 (0.8)	2.4 (0.6)	**0.007**	3.52 (0.3)	2.93 (0.5)	**<0.001**
83	3.32 (0.6)	2.78 (0.6)	**0.01**	3.35 (0.7)	2.67 (0.7)	**0.003**	3.5 (0.4)	3.16 (0.6)	**0.03**
117	3.4 (0.7)	2.81 (0.7)	**0.009**	3.41 (0.5)	2.74 (0.7)	**0.001**	3.5 (0.4)	3.4 (0.4)	0.424
267	3.48 (0.4)	3.12 (0.5)	**0.018**	3.42 (0.6)	3.02 (0.5)	**0.025**	3.55 (0.2)	3.44 (0.4)	0.271

**YOUNG**									
	F	N	*p*	H	N	*p*	H	F	*p*

17	1.35 (0.9)	1.24 (0.9)	0.202	1.62 (1)	1.24 (0.9)	**0.004**	1.62 (1)	1.35 (0.9)	**0.035**
33	1.35 (0.7)	1.26 (0.8)	0.584	1.58 (0.8)	1.26 (0.8)	0.068	1.58 (0.8)	1.35 (0.7)	0.105
50	3 (0.7)	2.85 (0.9)	0.227	3.03 (0.7)	2.85 (0.9)	0.158	3.03 (0.7)	3 (0.7)	0.872
67	3 (0.7)	3.03 (0.8)	0.354	3.52 (0.3)	3.03 (0.8)	**0.003**	3.52 (0.3)	3 (0.7)	**<0.001**
83	3.32 (0.6)	3.35 (0.7)	0.601	3.5 (0.4)	3.35 (0.7)	0.173	3.5 (0.4)	3.32 (0.6)	0.061
117	3.4 (0.7)	3.41 (0.5)	0.886	3.5 (0.4)	3.41 (0.5)	0.083	3.5 (0.4)	3.4 (0.7)	0.310
267	3.48 (0.4)	3.42 (0.6)	0.346	3.55 (0.2)	3.42 (0.6)	0.241	3.55 (0.2)	3.48 (0.4)	0.448

**OLD**									
	F	N	*p*	H	N	*p*	H	F	*p*

17	0.32 (0.4)	0.07 (0.4)	**0.005**	0.57 (0.5)	0.07 (0.4)	**<0.001**	0.57 (0.5)	0.32 (0.4)	0.087
33	0.53 (0.4)	0.18 (0.5)	**0.001**	0.71 (0.6)	0.18 (0.5)	**<0.001**	0.71 (0.6)	0.53 (0.4)	0.175
50	1.83 (0.8)	1.57 (0.8)	0.051	1.99 (0.9)	1.57 (0.8)	**<0.001**	1.99 (0.9)	1.83 (0.8)	0.287
67	2.5 (0.7)	2.4 (0.6)	0.273	2.93 (0.5)	2.4 (0.6)	**<0.001**	2.93 (0.5)	2.5 (0.7)	**0.013**
83	2.78 (0.6)	2.67 (0.7)	0.176	3.16 (0.6)	2.67 (0.7)	**0.007**	3.16 (0.6)	2.78 (0.6)	**0.021**
117	2.81 (0.7)	2.74 (0.7)	0.124	3.4 (0.4)	2.74 (0.7)	**<0.001**	3.4 (0.4)	2.81 (0.7)	**<0.001**
267	3.12 (0.5)	3.02 (0.5)	0.073	3.44 (0.4)	3.02 (0.5)	**<0.001**	3.44 (0.4)	3.12 (0.5)	**0.01**

*Y, young; O, old; F, fearful; N, neutral; H, happy. Bold p-values indicate significance level of <0.05*.

#### Response bias

A similar ANOVA of response bias (*C*) revealed effects of age, *F*(1, 36) = 41.75, *p* < 0.001, facial emotion, *F*(2, 72) = 40.18, *p* < 0.001, and an age by emotion interaction, *F*(2, 72) = 15.57, *p* < 0.001. In general, participants were less willing to respond that an expression was fearful or happy (i.e., conservative bias, *C* > 0) compared to respond that an expression was neutral (i.e., liberal bias, *C* < 0). This effect was stronger for older than younger participants.

#### Recognition performance: age and neuroticism

A univariate ANOVA with masked recognition performance (averaged across durations) as dependent variable and age, sex, and neuroticism (high, low) as fixed factors was computed. As showed in Figure 4, an interaction effect between age and neuroticism, *F*(1, 31) = 5.17, *p *= 0.030, indicates that across durations, high levels of neuroticism seem to have an opposite effect on performance in younger and older adults. A high level of neuroticism seems to be disadvantageous for younger, but not older adults. However, follow-up *t*-tests did not show differences between high and low neurotics in younger, *t*(17) = 1.79, *p* = 0.099, or older, *t*(18) = 1.01, *p* = 0.327, adults. Rather, the interaction seemed to be driven by age-related differences in low neurotic subjects, *t*(18) = 6.62, *p *< 0.001, but not in high neurotic subjects, *t*(17) = 1.81, *p* = 0.091, indicating a reduction in performance in young high, but not old high neurotics, compared to their low neurotic counterparts.

## Discussion

In the present study, visibility was parametrically reduced in order to investigate the temporal development of a happy faces advantage (e.g., Calvo and Lundqvist, 2008) in younger and older adults. Also, the combined effect of age and neuroticism, and its relation to arousal and performance was investigated.

In their meta-analysis of emotion recognition and aging, Ruffman et al. (2008) showed that younger adults performance were better than older adults for all expressions except disgust. But the age difference was smaller for happiness (and disgust) than for all other facial expressions. Our results from the unmasked recognition task mirror the findings in this meta-analysis, at least for neutral, fearful, and happy facial expressions. Although younger adults performance was better than older adults across all emotions, the age-related differences were smaller for happy than fearful faces. Our results are further expending those by Ruffman et al. (2008) by replicating the same recognition pattern with backwardly masked faces. As in the unmasked recognition task, younger adults performed better than older adults across expressions, but the age-related differences were smaller for happy than fearful and neutral faces. This shows that across age, happy faces have an advantage in recognition performance that holds even under restricted perceptual conditions (i.e., by the use of backwardly masked faces). Note however that neither our results, nor the results from the meta-analysis (Ruffman et al., 2008) support a so-called positivity effect among older adults, that is, a tendency for older adults to have a positivity bias in emotional attention and memory (Mather and Carstensen, 2005). As Ruffman et al. (2008) suggested, ceiling effects might explain why the effect size for the difference in performance on happy faces compared to other emotions was small. The present findings, both from the masked and the unmasked recognition tasks, clearly show that this was true for the younger adults also in our study. The only support for a positivity effect among older adults in our study is the findings that performance was better for happy than fearful and neutral faces at almost all durations for older, but not for younger adults. Since the task is perceptually demanding, these results may suggest that older adults rely on salient facial features to a greater extent than younger adults. In support for such a saliency-based explanation is the finding that older, but not younger adults’ masked recognition performance was better for fearful than neutral faces. However, this is inconsistent with the idea of neglecting negative information among older adults (e.g., Mather and Carstensen, 2005). Also inconsistent with the notion of a positivity effect in older adults, and more in line with a more general age independent happy face advantage, is the finding that across age, and for both the masked and the unmasked recognition tasks, happy faces were recognized better than fearful or neutral faces. Thus, instead of an age by emotion interaction, driven by age differences in recognition of negative, but not positive faces (e.g., Calder et al., 2003), inspection of our masked recognition results (Figure 3) rather indicate a similar performance trajectory across age for both negative and positive faces.

Our results show that in both age groups, recognition was better for happy than neutral and fearful faces across all durations. These findings are in line with previous studies with younger adults showing a happy face advantage primarily in visual attention (Juth et al., 2005; Calvo and Nummenma, 2008; Calvo and Marrero, 2009) but also in recognition (e.g., Calvo and Lundqvist, 2008). When younger adults were showed unmasked faces at fixed-display time, Calvo and Lundqvist (2008) found that recognition of happy faces reached ceiling levels at 50 ms duration. They also found that recognition performance was better for happy than all other expressions already at their shortest duration (25 ms). Thus, an investigation of the temporal development of a happy face advantage based on their results is difficult. By using a backward masking paradigm and SOAs ranging from 17 to 267 ms, our study adds to the literature by showing that at durations longer than 50 ms, both age groups recognized masked happy faces better than neutral and fearful faces. This might indicate that when more time is given, which presumably would allow more elaborative based processing, the more effective is the processing of happy faces compared to fearful and neutral faces. This, in turn, suggests that the happy face advantage is not merely a result of salient featural processing. In fact, as durations become longer, the masked recognition performance resembles the results from the unmasked recognition task. In other words, with increased visibility, emotion recognition may rely less on salient features, and instead shift to a more elaborative, emotional evaluation of the face. Although the present study cannot conclude when such a shift may occur on this continuum, this is of interest for future studies to explore. Further, younger adults reached the performance ceiling when happy faces were presented for 67 ms. This emphasizes the strength of the happy face advantage not only in spatial attention, as has been shown in visual search tasks (Juth et al., 2005; Calvo and Nummenma, 2008; Calvo and Marrero, 2009), but also in the temporal aspect of the processing of facial expressions. The finding that older adults reached ceiling for happy faces at the 117 ms duration demonstrate that the happy face advantage remains intact with age.

Our findings from the masked recognition task indicate that salient facial features might guide attention during early processing of facial expressions in reduced visible stimuli conditions. For instance, a smiling face (happy) reveals more white parts (teeth) around the mouth than a fearful or a neutral face does, which might facilitate salient feature processing (Calvo and Marrero, 2009). Especially when tasks require fast processing, participants may rely heavily on salient features. Such interpretations have recently been suggested in studies using visual search (Calvo and Nummenma, 2008) and attentional blink tasks (Miyazawa and Iwasaki, 2010), and this may account for the happy face advantage in early processing of facial expressions. However, a majority of the studies investigating the happy face advantage focus mainly on the perceptual part and do not thoroughly consider the emotional aspect. Before any of these explanations can be established, both aspects must be taken into consideration, preferably in the same study. Our combined masked and unmasked recognition results suggest a combined effect of processing of salient perceptual features and more elaborative processing in emotional facial expression recognition. Results from our unmasked recognition task, in which unlimited time was given to the participants, recognition performance was better for happy faces than neutral and fearful faces. These results are difficult to explain simply in terms of saliency or perception. On the other hand, the perceptually demanding backward masking task force participants to rely more on processing of salient features. However, it would be unrealistic to assume that this will be so also in a non-perceptually demanding task such as our unmasked recognition task. Rather, this task would tap on participants’ elaborative processing of the facial expression. In order to categorize the face into an emotional expression, the participants’ most likely need to evaluate the face and choose a label that fits their internal representation of that specific expression. Thus, when visibility is reduced, participants might rely more on salient features that match their internal representation of a facial expression. On the other hand, when the visibility is not reduced and enough time is given, the participants’ internal representation (i.e., labeling) should be a result of their elaborative processing of the face. From that point of view, our results from the masked recognition task would suggest a shift from salience based processing (at the shortest durations) to a more elaborative based processing (at the longest durations). If our masked recognition results are studied in light of a processing continuum from more salient to more elaborative processing, that would suggest that the happy face advantage emerge from salient based processing and extends to, and accelerates when reaching a more elaborative processing. That is, although happy faces were better recognized already at the shortest durations, this advantage was even more pronounced at the longer durations although our results from the masked and unmasked recognition task suggest a combined effect of processing of salient perceptual features and elaborative processing as explanatory factors for the happy face advantage, future studies most try to disentangle these factors within the same study-paradigm in order to investigate their individual contribution to the happy face advantage.

The backward masking procedure has been used extensively in functional magnetic resonance imaging studies to investigate amygdala activity in unconscious processing of facial expressions (e.g., Whalen et al., 1998; Duan et al., 2010). The detection of briefly exposed fearful faces have been showed to remain intact even in patients with bilateral (Tsuchiya et al., 2009) and left lateral (Palermo et al., 2010) amygdala lesions. Interestingly, amygdala lesions impair the ability to consciously recognize a fearful facial expression (Tsuchiya et al., 2009; Palermo et al., 2010). Such findings support the idea of functionally separated pathways for conscious and unconscious fear processing (see Tamietto and de Gelder, 2010 for a review). Whereas conscious processing of fear seems to rely on a cortical route including fusiform gyrus and the temporal pole, unconscious fear processing instead may depend on a subcortical route including the superior colliculus and the pulvinar (Morris et al., 1999). Because of its evolutionary relevance, fearful faces have received the most attention in imaging studies on amygdala activation to masked faces (e.g., Ottaviani et al., 2012). However, Williams et al. (2004) found that the amygdala was activated not only in response to fearful faces but also to happy faces during binocular suppression conditions, which might indicate that the information that reach amygdala through a subcortical route is not sufficient to discriminate between expressions. This is consistent with the assumption that the subcortical route’s role is to pass on low spatial rather than high spatial frequency visual information to amygdala (Vuillumier et al., 2003). In other words, without high spatial frequency information (e.g., sharp contours), the amygdala may only be able to do a rough evaluation of the face. Not until high spatial frequency information reaches the amygdala through the cortical route, a more detailed facial expression discrimination may occur (Vuillumier et al., 2003). Bar et al. (2006) showed that this bottom-up low spatial frequency information might, via a fast and crude pathway to the orbitofrontal cortex, activate an anticipation, which is subsequently matched with the accumulated information received from the slower cortical route. Similar “predictive codes” for faces stored in medial frontal cortex (MFC) are assumed to be matched with incoming information in order to confirm face recognition (Summerfield et al., 2006). These top-down facilitation models would be at least partially consistent with our masked recognition results in the sense that a happy face consists of more perceptual salient features (e.g., Calvo et al., 2010) which in turn might activate “stronger” anticipations compared to neutral and fearful faces. These differences in top-down activated anticipations would subsequently lead to differences in recognition performance between the facial expressions. In the light of such approach, the present masked recognition results would suggest a stronger and/or faster relation between a face template in MFC and confirmatory visual information for happy compared to neutral and fearful faces. Although the present behavioral study for obvious reasons cannot conclude whether any predictive facial expression templates exist and to what extent they might differ among expressions, this is of interest for future studies to explore.

Finally, the present study investigated the combined effect of age and neuroticism on facial expression recognition at different presentation durations. Our results revealed an interaction between age and neuroticism, indicating that being high in neuroticism might be disadvantageous for younger, but not older adults. The former is in line with previous findings showing decreased performance for high neurotic younger adults compared to low neurotic younger adults in attention demanding tasks (Szymura and Wodniecka, 2003). Our results extend these findings by suggesting reversed performance for older neurotic adults, at least during recognition of facial expressions. These data indicate increased performance for high neurotic older adults compared to low neurotic older adults. Current findings are also in the expected direction given previous findings of age-related differences in physiological arousal when exposed to emotional stimuli (Tsai et al., 2000; Gavazzeni et al., 2008). So, enhanced arousal from neuroticism in elderly persons could be beneficial to compensate for decreased arousal in older adults when exposed to emotional faces. Unfortunately, arousal was not directly measured in the present study, and therefore, we can only assume that level of arousal is mediating the interaction effect between age and neuroticism. However, as the age by neuroticism interaction was only evident in the briefly exposed masked recognition task and not in the self-paced intensity and emotion rating tasks, the findings support the interpretation that the effect is mediated by age-related arousal differences. Also, there was no interaction between facial emotion and neuroticism, which further emphasizes the general arousal assumption. There were no significant differences between high and low neurotics within age groups, which may be a result of the small sample size. For example, the study of Szymura and Wodniecka (2003) included sample sizes ranging between 64 and 102 participants; the current study included 39 participants. Nonetheless, the age by neuroticism interaction remain significant, but given the small sample size in the present study, these results are preliminary and needs to be replicated in a larger sample.

To summarize, in line with previous research on overt facial recognition (Calvo and Lundqvist, 2008), our results indicate that even when emotional faces are briefly exposed, and backwardly masked, younger and older adults’ recognition performance was better for happy than fearful or neutral faces. This happy face advantage is suggested to emerge from a combination of salient based and more elaborative processing of the happy face. Results from our two tasks showed that this advantage was also present in older adults, indicating a more general advantage for happy faces that is independent of age. Our results also revealed an interaction between age and neuroticism, showing that being high in neuroticism might be disadvantageous for younger but not older adults in arousal enhancing tasks.

## Conflict of Interest Statement

The authors declare that the research was conducted in the absence of any commercial or financial relationships that could be construed as a potential conflict of interest.

## References

[B1] Bar M. Kassam K. S. Ghuman A. S. Boshyan J. Schmid A. M. Dale M. S. (2006). Top-down facilitation of visual recognition. Proc. Natl. Acad. Sci. U.S.A. 103, 449–454 10.1073/pnas.0507062103 16407167 PMC1326160

[B2] Bornemann B. Winkielman P. van der Meer E. (2012). Can you feel what you do not see? Using internal feedback to detect briefly presented emotional stimuli. Int. J. Psychophysiol. 85, 116–124 10.1016/j.ijpsycho.2011.04.007 21571012

[B3] Bridgeman B. (1998). Durations of stimuli displayed on video display terminals: (n-1)/f plus persistence. Psychol. Sci. 9, 232–233 10.1111/1467-9280.00045

[B4] Calder A. J. Keane J. Manly T. Sprengelmeyer R. Scott S. Nimmo-Smith I. (2003). Facial expression recognition across the adult life span. Neuropsychologia 41, 195–202 10.1016/S0028-3932(02)00149-5 12459217

[B5] Calvo M. G. Lundqvist D. (2008). Facial expressions of emotion (KDEF): identification under different display-duration conditions. Behav. Res. Methods 40, 109–115 10.3758/BRM.40.1.109 18411533

[B6] Calvo M. G. Marrero H. (2009). Visual search for emotional faces: the role of affective content and featural distinctiveness. Cogn. Emot. 23, 782–806 10.1080/02699930802151654

[B7] Calvo M. G. Nummenma L. (2008). Detection of emotional faces: salient physical features guide effective visual search. J. Exp. Psychol. Gen. 137, 471–494 10.1037/a0012771 18729711

[B8] Calvo M. G. Nummenma L. Avero P. (2010). Recognition advantage of happy faces in extrafoveal vision: featural and affective processing. Vis. Cogn. 18, 1274–1297 10.1080/13506285.2010.481867

[B9] Costa P. T. McCrae R. R. (1985). The NEO Personality Inventory Manual. Odessa, FL: Psychological Assessment Resources

[B10] Costa P. T. McCrae R. R. (1992). Revised NEO Personality Inventory (NEO-PI-R) and NEO Five Factor Inventory (NEO-FFI) Professional Manual. Odessa, FL: Psychological Assessment Resources

[B11] Duan X. Dai Q. Gong Q. Chen H. (2010). Neural mechanism of unconscious perception of surprised facial expression. Neuroimage 52, 401–407 10.1016/j.neuroimage.2010.04.021 20398771

[B12] Dureman I. (1960). SRB:1. Stockholm: Psykologiförlaget

[B13] Ekman P. Friesen W. (1976). Pictures of Facial Affect. Palo Alto: Consulting Psychologists Press

[B14] Eysenck H. J. Eysenck M. W. (1985). Personality and Individual Differences: A Natural Science Approach. New York: Plenum Press

[B15] Folstein M. F. Folstein S. E. McHugh P. R. (1975). “Mini-mental state.” A practical method for grading the cognitive state of patients for clinician. J. Psychiatry Res. 12, 189–198 10.1016/0022-3956(75)90026-6 1202204

[B16] Gavazzeni J. Wiens S. Fischer H. (2008). Age effects to negative arousal differ for self-report and electrodermal activity. Psychophysiology 45, 148–151 17850240 10.1111/j.1469-8986.2007.00596.x

[B17] Hoffman H. Kessler H. Eppel T. Rukavina S. Traue H. C. (2010). Expression intensity, gender and facial emotion reognition: women recognize only subtle facial emotions better than men. Acta Psychol. (Amst.) 135, 278–283 10.1016/j.actpsy.2010.07.012 20728864

[B18] Humphreys M. S. Revelle W. (1984). Personality, motivation, and performance: a theory of the relationship between individual differences and information processing. Psychol. Rev. 91, 153–184 10.1037/0033-295X.91.2.153 6571423

[B19] Isaacowitz D. M. Löckenhoff C. E. Lane R. D. Wright R. Sechrest L. Riedel R. (2007). Age differences in recognition of emotion in lexical stimuli and facial expressions. Psychol. Aging 22, 147–159 10.1037/0882-7974.22.1.147 17385991

[B20] Juth P. Lundqvist D. Karlsson A. Öhman A. (2005). Looking for foes and friends: perceptual and emotional factors when finding a face in the crowd. Emotion 5, 379–395 10.1037/1528-3542.5.4.379 16366743

[B21] MacMillan N. A. Creelman C. D. (1990). Response bias: characteristics of detection theory, threshold theory, and nonparametric indexes. Psychol. Bull. 107, 401–413 10.1037/0033-2909.107.3.401

[B22] MacMillan N. A. Creelman C. D. (1991). Detection Theory: A User’s Guide. New York: Cambridge University Press

[B23] Mather M. Carstensen L. L. (2005). Aging and motivated cognition: the positivity effect in attention and memory. Trends Cogn. Sci. (Regul. Ed.) 9, 496–502 10.1016/j.tics.2005.07.010 16154382

[B24] McDowell C. L. Harrison D. W. Demaree H. A. (1994). Is right hemisphere decline in the perception of emotion a function of aging? Int. J. Neurosci. 79, 1–11 7744545 10.3109/00207459408986063

[B25] Miyazawa S. Iwasaki S. (2010). Do happy faces capture attention? The happiness superiority effect in attentional blink. Emotion 10, 712–716 10.1037/a0019348 21038954

[B26] Morris J. S. Öhman A. Dolan R. J. (1999). A subcortical pathway to the right amygdala mediating “unseen” fear. Proc. Natl. Acad. Sci. U.S.A. 96, 1680–1985 10.1073/pnas.96.4.1680 9990084 PMC15559

[B27] Ottaviani C. Cevolani D. Nucifora V. Borlimi R. Agati R. Leonardi M. (2012). Amygdala responses to masked and low spatial frequancy fearful faces: a preliminary fMRI study in panic disorder. Psychiatry Res. 203, 159–165 10.1016/j.pscychresns.2011.12.010 22944369

[B28] Palermo R. Schmalzl L. Mohamed A. Bleasel A. Miller L. (2010). The effect of unilateral amygdala removals on detecting fear from briefly presented backward-masked faces. J. Clin. Exp. Neuropsychol. 32, 123–131 10.1080/13803390902821724 19381996

[B29] Phillips L. H. MacLean R. D. J. Allen R. (2002). Aging and the perception and understanding of emotions. J. Gerontol. B Psychol. Sci. Soc. Sci. 57, 526–530 10.1093/geronb/57.6.P526 12426435

[B30] Ruffman T. Henry J. D. Livingstone V. Phillips L. H. (2008). A meta-analytic review of emotion and aging: implications for neuropsychological models of aging. Neurosci. Biobehav. Rev. 32, 863–881 10.1016/j.neubiorev.2008.01.001 18276008

[B31] Snodgrass J. G. Corwin J. (1988). Pragmatics of measuring recognition memory: applications to dementia and amnesia. J. Exp. Psychol. Gen. 117, 34–50 10.1037/0096-3445.117.1.34 2966230

[B32] Summerfield C. Egner T. Greene M. Koechlin E. Mangels J. Hirsch J. (2006). Predictive codes for forthcoming perception in the frontal cortex. Science 314, 1311–1314 10.1126/science.1132028 17124325

[B33] Szymura B. Wodniecka Z. (2003). What really bothers neurotics? In search for factors impairing attentional performance. Pers. Individ. Dif. 34, 109–126 10.1016/S0191-8869(02)00034-X

[B34] Tamietto M. de Gelder B. (2010). Neural bases of the non-conscious perception of emotional signals. Nat. Rev. Neurosci. 11, 697–709 10.1038/nrg2844 20811475

[B35] Tsai J. L. Levenson R. W. Carstensen L. L. (2000). Autonomic, subjective, and expressive responses to emotional films in older and younger Chinese Americans and European Americans. Psychol. Aging 15, 684–693 10.1037/0882-7974.15.4.684 11144327

[B36] Tsuchiya N. Moradi F. Felsen C. Yamazaki M. Adolphs R. (2009). Intact rapid detection of fearful faces in the absence of the amygdala. Nat. Neurosci. 12, 1224–1225 10.1038/nn.2380 19718036 PMC2756300

[B37] Vuillumier P. Armony J. L. Driver J. Dolan R. J. (2003). Distinct spatial frequency sensitivities for processing faces and emotional expressions. Nat. Neurosci. 6, 624–631 10.1038/nn1057 12740580

[B38] Whalen P. J. Rauch S. L. Etcoff N. L. McInerney S. C. Lee M. B. Jenike M. A. (1998). Masked presentation of emotional facial expressions modulate amygdala activity without explicit knowledge. J. Neurosci. 18, 411–418 9412517 10.1523/JNEUROSCI.18-01-00411.1998 PMC6793390

[B39] Wiens S. Fransson P. Dietrich T. Lohmann P. Ingvar M. Öhman A. (2004). Keeping it short: a comparison of methods for brief picture presentation. Psychol. Sci. 15, 282–285 10.1111/j.0956-7976.2004.00667.x 15043649

[B40] Williams M. A. Morris A. P. McGlone F. Abbott D. F. Mattingley J. B. (2004). Amygdala responses to fearful and happy facial expressions under conditions of binocular suppression. J. Neurosci. 24, 2898–2904 10.1523/JNEUROSCI.4977-03.2004 15044528 PMC6729857

[B41] Yerkes R. M. Dodson J. D. (1908). The relation of strength of stimulus to rapidity of habit-formation. J. Comp. Neurol. 18, 459–482 10.1002/cne.920180503

